# Factorial validity and measurement invariance of a self-reported scale of paradoxical leadership behaviours: evidence from sport industry leaders

**DOI:** 10.3389/fpsyg.2025.1541891

**Published:** 2025-02-21

**Authors:** Matthew Bourke, John Cairney, Veronique Richard, Samantha Mulcahy, Chris Moos, Sue Dopson

**Affiliations:** ^1^Health and Wellbeing Centre for Research Innovation, School of Human Movement and Nutrition Sciences, The University of Queensland, Brisbane, QLD, Australia; ^2^Saïd Business School, Social Sciences Division, University of Oxford, Oxford, England, United Kingdom

**Keywords:** paradoxes, leadership style, factorial validity, self-report, sport industry

## Abstract

**Introduction:**

Organisational leaders across all sectors are often faced with a dynamic, unpredictable and complex business landscape. Understanding leadership style is integral to optimising leadership and organisational culture and performance. One such leadership style that warrants further investigation is Paradoxical Leadership. This study examines the factorial validity and measurement invariance of a self-report version of the Paradoxical Leadership Scale (PLS) among a diverse sample of sport industry leaders. These sport industry leaders (*n* = 345) provided a platform to explore paradoxical leadership in a seldom-examined sector.

**Methods:**

Participants were recruited through an online campaign, partnering with the sports media company, SportsPro. Factorial validity of the self-reported PLS was examined using Exploratory Structural Equation Modelling (ESEM), and measurement invariance testing was conducted using Multiple Indicator Multiple Causes (MIMIC) modelling across demographic and contextual factors.

**Results:**

Results confirmed the second-order five factor model as the best fit, with partial deviations in item loadings but maintaining overall structural integrity. The scale showed full invariance across managerial levels, experience, gender, and regions, with partial invariance across age groups, indicating its robust applicability.

**Discussion:**

This is the first study to explore the factorial validity and measurement invariance of a modified, self-report scale measuring paradoxical leadership behaviours in a diverse sample of sport leaders. The findings support the use of this scale for both research and practical application in this context. Further research applying this scale across sectors is recommended.

## Introduction

In today’s dynamic and unpredictable business landscape, organisations grapple with volatile, uncertain, complex, and ambiguous (VUCA) environments (Bennis and Naus, 1985) while also contending with brittle, anxious, nonlinear, and incomprehensible (BANI) challenges that emphasise internal fragility and the psychological impact of constant change (Vishwakarma and Vishwakarma, 2024). Such environments breed complexity, prompting organisational systems to adapt and evolve continuously. Within these systems, elements often coexist in contradiction, leading to the emergence of paradoxes that challenge traditional notions of decision-making and management (Smith and Lewis, 2011). These paradoxes manifest as “persistent contradictions between interdependent elements” (Schad et al., 2016, p. 10). In this sense, organisational paradoxes are not only about contradiction. They involve inter-dependencies and tend to persist over time. Paradoxes manifest at both macro and micro levels within organisations and encompass various domains such as learning, belonging, organising, and performing (Lewis and Smith, 2022; Smith and Lewis, 2011). For instance, organisations may confront paradoxes regarding the pace of innovation, conflicting values, the balance between control and flexibility (e.g., Aoki, 2019; Wareham et al., 2014), or the trade-off between profit and social responsibility (Andriopoulos and Lewis, 2009).

Leaders capable of balancing opposing demands and managing complex inter-dependencies are better equipped to navigate VUCA and BANI environments. Managing paradox inside organisations requires paradoxical leadership (Lewis, 2000). This leadership style is associated with several positive organisational outcomes including organisational performance (Amason, 1996), employee performance (Zhang et al., 2014), innovation (Zhang et al., 2022) and creativity (Yang et al., 2021).

Zhang et al. (2014) proposed a comprehensive framework for understanding paradoxical leadership behaviours, delineating five key dimensions: self-and other-centeredness (i.e., the degree to which a leader is the centre of influence while sharing recognition with others), distance and closeness (i.e., maintaining hierarchy with regards to work issues while simultaneously forming close personal bonds), uniformity and individualization (i.e., treating all subordinates equally while prioritising their individual skills or interests), enforcement and flexibility (i.e., enforcing work requirements while allowing for flexibility in how work is done), and control and autonomy (i.e., maintaining control over strategic decisions while allowing subordinates to manage smaller details). This model has since become a cornerstone in the study of paradoxical leadership, guiding research and assessment in this field. Research using this measure has demonstrated positive association between managers’ paradoxical leadership behaviours with employees’ job-related performance, job related attitudes and perceptions, and favourable perceptions of their manager (Lee et al., 2023).

## Measuring paradoxical leadership behaviours

The original paper (Zhang et al., 2014), and subsequent studies (see Lee et al., 2023), have relied on subordinate reporting to measure paradoxical leadership behaviours; in other words, employees’ rate or assess the paradoxical leadership behaviours of their supervisors or managers. This approach is common in organisational behaviour research and is employed, in part, to overcome several self-reporting biases (e.g., social desirability bias, self-serving bias, lack of awareness). However, there are many circumstances under which self-reported leadership behaviour measures are desirable, both in practical terms but also in research applications.

In research, understanding the leader’s own perceptions of their behaviours and leadership styles can be useful, especially when attempting to understand how other behaviours, traits and psychological characteristics of the leader may be associated with leadership style. For example, paradoxical leaders have been shown to exhibit extraversion and openness to experience (Ishaq et al., 2021), are holistic thinkers (Zhang et al., 2014), and possess a long-term orientation (Zhang and Han, 2019). A better understanding of the psychological factors associated with paradoxical leadership may assist in both supporting leaders and in the identification of leaders who practice paradoxical leadership behaviours (e.g., recruitment).

There are also circumstances where it may be desirable to examine alignment between leaders’ perceptions of their own leadership behaviours, against the perceptions of their subordinates (e.g., Aarons et al., 2017; Caniëls, 2023), or the overarching goals of the organisation. In order to compare leader and follower perceptions, both leader-and subordinate-reporting of leadership behaviours is required. Ensuring that both formats (self-versus employee-report scales) have comparable psychometric properties (i.e., that there are measuring the same underlying construct) is essential when studying specific leadership constructs.

Finally, it is not always feasible or practical to survey both leaders and their subordinates. A self-reported measure of paradoxical leadership behaviours allows for use in research contexts that may be constrained by resources or access to participants. In such circumstances, researchers may need to rely on leader-reported behaviours as the only marker of leadership style that is practically obtainable. Once again, it is critical that self-report measures are psychometrically aligned to the subordinate-reported measures.

Beyond research, the application of self-reported leadership behaviours measures in leadership and/or human resources management practice have several potential uses. Self-reports reveal a leader’s intended behaviours and self-awareness, showing how they aim to enact leadership (Hartung, 2020). Understanding leaders’ intentions offers a foundation for examining whether their actions align with organisational goals or leadership ideals (Nöthel et al., 2023; Rowe, 2001). Self-reported measures allow leaders to track their development over time, reflecting on areas they have worked to improve or change (Church and Rotolo, 2010). This perspective is crucial for professional development, as leaders need to recognise and acknowledge both their strengths and areas for improvement.

Self-reported behaviours also serve as an important foundation for 360-degree feedback processes (Nowack and Mashihi, 2012). Leaders’ self-assessments can be compared with employee feedback to identify gaps between self-perception and external perception, fostering deeper insights and promoting alignment between intended and perceived leadership behaviours (Ellison et al., 2022). Therefore, self-report tools have an important place in professional practice for self-assessment and self-reflection. Indeed, previous research has shown that leaders who engage in self-reflection are often more effective and empathetic (Lee and Jung, 2022), which can lead to improved team performance and morale (Ellison et al., 2022).

Interestingly, results on rating agreement have demonstrated that agreement is much stronger between ratings from others (e.g., the rating of a supervisor between multiple subordinates or peers) compared to self-other ratings (i.e., how a supervisor rates themselves compared to how they are rated by others; Conway and Huffcutt, 1997; Harris and Schaubroeck, 1988). This may demonstrate that there is unique measurement error between scales that are designed to assess others and scales that are designed to assess oneself which may distort the relationship between each of the rating scales (Murphy, 2008). As with self-report measures used in research, human resource specialists should be confident that the measures they are using inside organisations are psychometrically sound, accurately capturing what they purport to measure and can be confidently compared to assessments from other sources. The absence of any evidence on the psychometric properties of a self-reported PLS limits both researchers and practitioners from examining and/or attempting to understand and support these behaviours inside organisations.

### Cultural foundations and cross-cultural validation of paradoxical leadership

While self-reported paradoxical leadership behaviours offer valuable insights into personal development and professional practice, their application and interpretation can vary significantly across cultural contexts. This highlights the importance of exploring the cultural foundations of paradoxical leadership and the need for cross-cultural validation to ensure tools like the PLS are effective in diverse organisational settings.

Rooted in Eastern philosophies, particularly the concept of Ying and Yang (Li, 2016; Nisbett et al., 2001; Smith and Lewis, 2011), much of the research on paradoxical leadership has emerged from Eastern cultures, with the PLS being validated among managers and employees from Chinese organisations (Zhang et al., 2014). However, as research into paradoxical leadership rapidly expands, there is a growing need to cross-validate the PLS in other cultural contexts to enable robust research into paradoxical leadership behaviours on a global scale (Miron-Spektor et al., 2017). While Eastern philosophies embrace paradoxical frames and take holistic approaches to navigating paradoxes (Nisbett et al., 2001), Western philosophies often encourage a more compartmentalised approach, separating and interrogating components individually (Batool et al., 2023; Keller et al., 2017; Miron-Spektor et al., 2017). Consequently, managers’ approaches to integrating paradoxical leadership behaviours into their practices may vary between Eastern and Western cultures. This may also affect perceptions or self-awareness of leaders’ own paradoxical leadership behaviours. Despite these differences, there has been limited research into the validity of the PLS in Western cultures, and the research which has been done does not provide strong support of the use of the scale in Western organisations (Franken et al., 2020; Shi, 2018).

While validation of the PLS outside Eastern contexts is important for the current subordinate-reported version, it is not less important for modifications to that scale including self-report.

### Application of paradoxical leadership inside sport: a novel context

A potentially interesting domain for the application of self-reported paradoxical leadership behaviours—both in research and in practice—is inside the sport industry. This context is broad and diverse, containing many different organisations. For example, there is a broad distinction to be made between professional (for profit) and not-for-profit sporting organisations. The former subsumes professional clubs (e.g., Real Madrid, New York Yankees) across a wide array of different sports, but also the brand or rights holders (e.g., La Liga, Major League Baseball) of the leagues that govern teams. In the not-for-profit domain, there are clubs (e.g., sporting teams), and, depending on the context, various layers of organisational governance structures. For example, as in the case of Australia, a federated system organises sport into state and national bodies (e.g., Sotiriadou, 2009). At the club or team level, leadership can be examined within different roles such as player leadership, formal (team captain) and informal (role modelling), leadership of coaches and sport administrators (e.g., managers, directors). There are also the ancillary industries that support sport, particularly in the professional or for-profit contexts. Sport marketing, sports media and broadcasting, sports apparel and equipment, and sport tech are also examples of industries where leadership can be studied. Within each of these are organisational roles (C-suite, Directors, Managers, Supervisors), where leadership behaviours can be enacted.

Previous research in sport management have explored leadership behaviours and practices in coaches (e.g., Kim and Cruz, 2016), among players (e.g., Coker et al., 2022), and sport administers (e.g., Soucie, 1994), across a variety of different contexts. Diversity is further evident in the kinds of leadership behaviours that have been studied. Transformational leadership (Arthur et al., 2017), servant leadership (Robinson et al., 2018), social-identity leadership (Stevens et al., 2021), to name a few, have been the focus of inquiry and these too have been studies across different roles (e.g., coach, sport administrator) and organisational contexts.

Interestingly, paradoxical leadership has not been a focus of research in sport leadership. Yet, as noted above, this style of leadership is particularly well-suited to leadership in VUCA environments—the kind of environments that give rise to contradiction and paradox. In all aspects of the sports industry, multiple paradoxes arise as organisations strive to harmonise financial sustainability, sporting excellence, community engagement, and social impact (Isard et al., 2023; Raw et al., 2022). Paradoxes, such as balancing competition and cooperation (Babiak and Thibault, 2007), may also arise relating to interorganisational relationships in sport, for example between local and national sporting organisations (Zheng et al., 2019), or among sporting organisations with diverging motives (Alexander et al., 2008; Sotiriadou et al., 2017). Effectively managing these paradoxes is crucial for organisational success and longevity (Miron-Spektor et al., 2017; Smith and Lewis, 2011). Leaders play a pivotal role in navigating these complexities, requiring them to adopt a paradigm shift from a traditional either/or approach to a more nuanced both/and perspective (Lewis et al., 2014). Paradoxical leadership, characterised by the ability to reconcile seemingly contradictory priorities, facilitates agile decision-making and strategic adaptability across all levels of management (Lewis et al., 2014). At the same time, paradoxes often escalate to the top. Hence, the experience of dealing with paradoxes changes as leaders rise from organisational silos to more integrative and outward facing roles.

As previously discussed, although the existing subordinate-reported measure of paradoxical leadership behaviours (Zhang et al., 2014) can be applied in the sport industry for both research and human resource practices, the lack of a validated self-report measure poses similar limitations to those observed in other organisational contexts. As in other settings, the sport industry stands to benefit from a self-report tool, both for advancing research (e.g., examining discrepancies between leader and follower perceptions) and for practical applications (e.g., enhancing leadership development efforts). In relation to the latter, having a validated self-measure could prove very useful in supporting the development and training of paradoxical leadership inside the sport industry.

### Aims of this study

In this study, we address key gaps in the literature by examining some core psychometric properties of a modified self-report version of the PLS (Zhang et al., 2014). Specifically, we test the factor structure of the self-report version to confirm that it reflects the same underlying dimensions as the original subordinate-report scale. This validation is crucial for maintaining confidence in the scale’s ability to accurately measure paradoxical leadership behaviours, ensuring its reliability for both theoretical research and practical applications.

We also assess measurement invariance across diverse groups, including managerial levels, gender, years of experience, and geographical regions. Establishing invariance ensures that the scale functions consistently across these groups, enabling meaningful comparisons and enhancing its generalizability in cross-cultural and organisationally diverse settings. As noted above, self-report leadership scales have significant potential for research and practice. Ensuring the modified scale retains the original dimensions and operates reliably across varied contexts is essential for its validity and utility.

Finally, we examine these key properties in a sample of senior leaders drawn from a diverse range of organisations across the sport industry. As noted, paradoxical leadership has yet to be studied in the context of sport. Using a diverse sample of sport leaders for initial psychometric testing of a self-report measure lays the foundation for both further research and practical applications inside the sport industry.

*Hypothesis* 1. The modified self-report scale for paradoxical leadership behaviours will have the same underlying factor structure as the original, subordinate-reported version. Specifically, there will be a five-factor structure and items loadings on the modified version will mirror the original.

*Hypothesis* 2. The measure will be invariant across different managerial levels, gender, age, years of experience, and geographical regions.

## Methods

### Participants and procedures

Participants were recruited through an online recruitment campaign. Researchers partnered with the United Kingdon sports media company SportsPro^1^ to recruit senior and middle managers working in sports related industries across the globe to participate in this study. Although SportPro’s audience is international, a large share of their audience is based in Western Europe and North America. SportsPro launched the recruitment campaign in November 2023 and the campaign ran for 3 months, ending in January 2024. An online questionnaire (administered through Qualtrics Software [Provo, Utah, United States]) was distributed through a database of individuals employed in sports industries, with additional recruitment via SportsPro social media pages (i.e., LinkedIn). Participants were eligible if they were employed in a sports related industry (e.g., sports league, sporting club, service/technology provider, broadcaster), and they held a managerial position in that organisation (i.e., they had at least one subordinate who reported directly to them). Ethics approval was obtained through The University of Queensland Human Research Ethics Committee for this study. All participants provided informed consent to participate in this study. All participant responses were anonymised.

### Measures

#### Paradoxical leadership behaviours

Paradoxical leadership behaviours were measured using a modified version of the 22-item PLS (Zhang et al., 2014). The original scale was modified to assess self-reported perceptions or paradoxical leadership behaviours relating to oneself, rather than paradoxical leadership relating to another person. To achieve this, the stem of the question on the PLS was changed from “my supervisor” to “I” (e.g., *I* show a desire to lead, but allow others to share the leadership role). Additionally, modifications were made to the response options to assess participants perceptions of the extent to which they see themselves as a paradoxical leader, rather than the frequency at which they used paradoxical leadership behaviours. Therefore, participants were asked to rate how strongly they agree with the sentiment of the statement on a Likert scale from Strongly Disagree to Strongly Agree, rather than reporting how frequently they engage in each paradoxical leadership behaviour from Never to A lot as was assessed in the original PLS.

#### Participant characteristics

Participants were asked to complete a range of questions to assess their personal characteristics and the characteristics of their organisation. Personal characteristics included age, gender, years of experience in their current position, and their position within the organisation (upper [e.g., C level], middle/lower [e.g., team leader]). Characteristics of the organisation included the region in the world that the company is based (i.e., North America, Europe, Asia, Australia and New Zealand, Africa), and the type of organisation (e.g., league, club, service provider).

### Statistical analysis

The factorial validity of the self-reported PLS was assessed using Exploratory Structural Equation Modelling (ESEM; Asparouhov and Muthén, 2009). ESEM is an approach to examine factorial validity that combines the strengths of confirmatory factor analysis (CFA) and exploratory factor analysis (EFA). Specifically, consistent with EFA, ESEM allows for non-zero cross-loadings among factors (which is inherent in psychological measurement), however, consistent with CFA, it also allows for model-based testing of *a-priori* hypotheses based on partial knowledge of the factor structure through the use of target rotations (Browne, 2001; Marsh et al., 2014). ESEM is proven to provide less biased results than CFA (Marsh et al., 2009). For the present analysis, a range of factor structures were estimated, consistent with those tested by Zhang et al. (2014). These included a first-order *single factor* structure, where all items load onto a single overall paradoxical leadership factor; a first-order *three-factor* structure, which used an exploratory approach where hypothesised factor structures were not defined *a-priori* and the best fitting model was estimated; and a first-order *five-factor* model consistent to the model identified by Zhang et al. (2014). Additionally, a second-order factor model was estimated where all the first-order factors from the best-fitting model were loaded onto a higher-order factor of paradoxical leadership. Hierarchical models were estimated using ESEM-within-CFA, where first-order ESEM solutions can be re-expressed using CFA to estimate higher order factors. A range of fit statistics including the RMSEA, SRMR, CFI, and TLI were used to determine the best fitting model. Acceptable model fit was determined using the following fit statistics: RMSEA ≤0.06, SRMR ≤0.08 and CFI ≥ 0.95 (Hu and Bentler, 1999).

The multiple indicator multiple causes (MIMIC) approach was used to assess factorial invariance. This approach uses a regression model where latent variables are regressed on variables over which invariance is tested (Morin et al., 2016). The MIMIC approach to examining invariance has some important methodological advantages compared to the multiple-group approach (Marsh et al., 2014). Specifically, it does not require the estimation of a separate model for each group which is beneficial for studies with modest sample sizes, especially when there is an imbalance in group sizes. Additionally, MIMIC enables assessment of invariance across a continuous indicator (e.g., age, years of experience), or based on variable where there are a larger number of smaller groupings (e.g., region of the world). As described by Morin et al. (2016) the assumption of measurement invariance is tested through comparing the results from a series of nested models. In the first model (null model), the regression model is estimated with the effect of predictors on latent means and intercepts are constrained to zero. In the second model (saturated) the effect of the predictor on item intercepts is freed, but the effect of the predictor on the latent means is constrained to zero. Finally, in the third model (invariant) the item intercepts are constrained to be invariant across levels of a predictor, while the predictor variable is freed to influence latent means. When the results indicate that the fit of the saturated and invariant models are better than the null model, the predictors are assumed to influence the latent variables. In this scenario, the results of the saturated and invariant models are compared. If the saturated model fits the data substantively better than the invariant model, this indicates that there is differential item functioning, and the assumption of invariance is not met. The cut-off values suggested by Chen (2007) were applied to MIMIC to quantify “substantive” differences in favour of the saturated model compared to the invariant models (i.e., CFI up to 0.010 lower in the saturated model and the RMSEA is no greater than 0.015 higher in the saturated model). Partial invariance was tested if full invariance is not achieved by sequentially freeing the association between predictors and individual item intercepts in the invariant model until the assumption of invariance is met. Given the limitations of ESEM to assess hierarchical models, invariance testing was conducted for first-order factors only.

## Results

### Descriptive statistics

A total of 345 participants had valid data and were included in the analysis. The participants were, on average, 46.99 years old (SD = 11.56), the majority of these participants were men (*n* = 263, 76.2%) and worked in upper management (*n* = 212, 61.4%). Most participants worked for organisations based in Europe (*n* = 201, 58.3%) followed by North America (*n* = 69, 20.0%), Australia/New Zealand (*n* = 30, 8.7%), Asia (*n* = 25, 8.7%), Africa (*n* = 13, 3.8%) and South America (*n* = 7, 2.0%). Descriptive statistics for the PLS items are displayed in Table 1 and descriptive statistics for the subscales including reliability estimates and bivariate correlations between subscales and total PLS score are displayed in Table 2.

**Table 1:** Descriptive statistics of individual items from the paradoxical leadership scale.

Item	Mean	SD	Skewness	Kurtosis
1. I use a fair approach to treat all my subordinates uniformly, but I also treats them as individuals	5.34	0.91	−2.51	9.03
2. I put all my subordinates on an equal footing, but consider their individual traits or personalities	5.05	1.05	−1.65	3.52
3. I communicate with my subordinates uniformly without discrimination, but vary my communication style depending on the subordinate’s individual characteristics of needs	5.14	1.00	−1.67	3.76
4. I manage my subordinates uniformly, but consider their individual needs	4.97	1.05	−1.55	3.29
5. I assign equal workload, but considered subordinate’s individual strengths and capabilities to handle different tasks	4.52	1.21	−0.74	0.12
6. I show a desire to lead, but allow others to share the leadership role	4.94	0.95	−1.22	2.41
7. I like to be the centre of attention, but allow others to share the spotlight as well	3.54	1.34	−0.16	0.91
8. I insist on getting respect, but also show respect towards others	4.70	1.22	−1.05	0.85
9. I have a high self-opinion, but show awareness of the personal imperfection and the value of other people	4.35	1.19	−0.69	0.07
10. I am confident regarding personal ideas and beliefs, but acknowledge that I can learn from others	5.28	0.77	−1.50	4.98
11. I control important work issues, but allow subordinates to handle details	4.67	1.06	−1.18	1.82
12. I make final decisions for subordinates, but allow subordinates to control work specific issues	4.19	1.19	−0.36	−0.55
13. I make decisions about big issues, but delegate lesser issues to subordinates	4.68	1.05	−0.89	0.79
14. I maintain overall control, but give subordinates appropriate autonomy	5.03	0.87	−1.27	3.12
15. I stress conformity in task performance, but allow for exceptions	3.94	1.23	−0.46	−0.41
16. I clarify work requirements, but do not micro-manage work	5.10	0.87	−1.07	1.52
17. I am highly demanding regarding work performance, but I am not hypercritical	4.56	0.99	−0.74	0.65
18. I have high work requirement, but allow subordinates to make mistakes	4.88	0.88	−1.15	2.71
19. I recognise the distinction between supervisors and subordinates, but do not act superior in my leadership role	4.96	0.96	−1.03	1.17
20. I keep distance from subordinates, but do not remain aloof	3.40	1.32	−0.02	−0.86
21. I maintain position difference, but uphold subordinates’ dignity	4.10	1.32	−0.45	−0.56
22. I maintains distance from subordinates at work, but I am also amiable towards them	3.53	1.45	−0.01	−1.09

**Table 2:** Reliability estimates and correlation coefficients among modified paradoxical leadership subscales and total score.

	α	1	2	3	4	5
1. Uniformity/ Individualisation	0.84	–				
2. Self-centred/ Other-centred	0.63	0.20**	–			
3. Controlling/ Allowing autonomy	0.73	0.22**	0.41**	–		
4. Demanding/ Flexible	0.67	0.30**	0.20**	0.34**	–	
5. Distant/ Close	0.85	0.09	0.28**	0.33**	0.13*	–
6. Overall PLS	0.82	0.61**	0.62**	0.73**	0.61**	0.58**

*p < 0.05, **p < 0.001.

### Exploratory structural equation models

The results from the ESEM models are displayed in Table 3. Consistent with the original scale (Zhang et al., 2014), results showed that the second order five-factor model fit the data best. The item loadings on each factor are displayed in Table 4. Interestingly, there were a handful of items which loaded to different factors than proposed by the original PLS. Specifically, the items *“I show a desire to lead, but allow others to share the leadership role”* and “*I am confident regarding personal ideas and beliefs, but acknowledge that I can learn from others*” both primarily loaded onto the Demanding/Flexible factor rather than the Self-centred/Other-centred factor as originally proposed. The item *“I stress conformity in task performance, but allow for exceptions”* loaded primarily onto the Controlling/Allowing autonomy factor, not the Demanding/Flexible factor. Lastly, the item *“I recognize the distinction between supervisors and subordinates, but do not act superior in my leadership role”* loaded primarily onto the Demanding/Flexible factor than the Distant/Close factor as found in the original PLS. In relation to our first hypothesis, we found support for the proportion that the factor structure of the self-report measure would be comparable with the original, subordinate-reported version, but only partial support for the item loading level.

**Table 3:** Model fit statistics from the exploratory structural equation modelling (ESEM).

	RMSEA	SRMR	CFI	TLI
First order 1 factor	0.129	0.117	0.435	0.376
First order 3 factor	0.067	0.054	0.877	0.831
First order 5 factor	0.030	0.024	0.981	0.967
Second order 5 factor	0.027	0.024	0.984	0.973

**Table 4:** Results from the second order 5 factor exploratory structural equation model of the self-reported paradoxical leadership behaviour scale.

	1. Uniformity/Individualisation	2. Self-centred/Other-centred	3. Controlling/Allowing autonomy	4. Demanding/Flexible	5. Distant/Close
1. I use a fair approach to treat all my subordinates uniformly, but I also treats them as individuals	**0.725**	−0.005	0.051	0.116	0.004
2. I put all my subordinates on an equal footing, but consider their individual traits or personalities	**0.790**	−0.008	0.050	0.029	0.005
3. I communicate with my subordinates uniformly without discrimination, but vary my communication style depending on the subordinate’s individual characteristics of needs	**0.690**	0.110	0.037	0.169	−0.009
4. I manage my subordinates uniformly, but consider their individual needs	**0.797**	0.090	0.106	0.086	0.019
5. I assign equal workload, but considered subordinate's individual strengths and capabilities to handle different tasks	**0.571**	0.041	0.090	0.083	0.108
6. I show a desire to lead, but allow others to share the leadership role	0.185	0.035	0.106	**0.338**	0.048
7. I like to be the centre of attention, but allow others to share the spotlight as well	0.043	**0.514**	0.131	−0.022	0.163
8. I insist on getting respect, but also show respect towards others	0.137	**0.510**	0.266	0.093	0.160
9. I have a high self-opinion, but show awareness of the personal imperfection and the value of other people	0.120	**0.588**	0.240	0.123	0.061
10. I am confident regarding personal ideas and beliefs, but acknowledge that I can learn from others	0.186	0.219	0.233	**0.384**	−0.006
11. I control important work issues, but allow subordinates to handle details	0.097	0.237	**0.542**	0.160	0.057
12. I make final decisions for subordinates, but allow subordinates to control work specific issues	0.111	0.285	**0.571**	0.001	0.209
13. I make decisions about big issues, but delegate lesser issues to subordinates	0.020	0.009	**0.728**	0.117	0.130
14. I maintain overall control, but give subordinates appropriate autonomy	0.111	0.012	**0.537**	0.301	0.029
15. I stress conformity in task performance, but allow for exceptions	0.122	0.102	**0.393**	0.020	0.182
16. I clarify work requirements, but do not micro-manage work	0.114	−0.141	0.161	**0.488**	0.043
17. I am highly demanding regarding work performance, but I am not hypercritical	−0.036	0.105	0.276	**0.477**	0.099
18. I have high work requirement, but allow subordinates to make mistakes	0.099	0.021	−0.007	**0.643**	0.020
19. I recognize the distinction between supervisors and subordinates, but do not act superior in my leadership role	0.189	0.033	0.018	**0.540**	−0.025
20. I keep distance from subordinates, but do not remain aloof	0.002	0.079	0.113	0.106	**0.811**
21. I maintain position difference, but uphold subordinates’ dignity	0.083	0.133	0.232	0.024	**0.689**
22. I maintains distance from subordinates at work, but I am also amiable towards them	0.021	0.053	0.091	−0.023	**0.881**

Bolded values indicate the strongest factor loading for each item.

### Measurement invariance

Results from the MIMIC measurement invariance testing are displayed in Table 5. Results demonstrated that the self-reported PLS was fully invariant among managerial levels (i.e., upper vs. middle/lower), years of experience in a position, gender, and geographical regions (i.e., North America, Europe, Asia, Australia/New Zealand, Africa). Additionally, the self-reported PLS scale has partial invariance across ages. Specifically, the association between age and item means for “*I am confident regarding personal ideas and beliefs, but acknowledge that I can learn from others”* and *“I make final decisions for subordinates, but allow subordinates to control work specific issues”* were freed, with item intercept of the first item being greater in older participants and the mean of the second item being greater in younger participants. Therefore, our second hypothesis was supported with regard to position, gender and geographical region, but only partially supported with regard to age.

**Table 5:** Results from the multiple indicator multiple causes (MIMIC) assessment of measurement invariance.

	RMSEA	∆ RMSEA	CFI	∆CFI
Managerial level				
Null model	0.035	–	0.970	–
Saturated model	0.028	−0.007	0.983	0.013
Invariance model	0.034	−0.001	0.973	0.003
Years experience				
Null model	0.031	–	0.976	–
Saturated model	0.030	−0.001	0.981	0.005
Invariance model	0.032	0.001	0.975	−0.001
Gender				
Null model	0.024	–	0.985	–
Saturated model	0.030	0.006	0.981	−0.004
Invariance model	0.025	0.001	0.984	−0.001
Age				
Null model	0.036	–	0.968	–
Saturated model	0.027	−0.009	0.985	0.017
Invariance model	0.037	0.001	0.967	−0.001
Partial invariance model	0.025	−0.011	0.986	0.018
Region				
Null model	0.031	–	0.967	–
Saturated model	0.030	−0.001	0.981	0.014
Invariance model	0.029	−0.002	0.973	0.008

## Discussion

The primary aim of this study was to assess the factorial validity and measurement invariance of a self-reported PLS evaluating managers’ self-perceptions of paradoxical leadership behaviours applied within sports-related organisations globally. The results indicate that the original second-order model with five lower-order factors fits the data well, albeit with some individual items loading onto different factors than initially proposed. Furthermore, our findings demonstrate that the factorial structure of the self-reported PLS remains invariant across multiple individual and contextual factors. This demonstrates that this scale is robust and is likely to have applicability across diverse managerial and organisational contexts.

This is the first study to provide support for the use of a self-reported assessment of paradoxical leadership style, and support for using the PLS to assess paradoxical leadership outside of Eastern cultures. However, there were some important nuances in the factorial structure of the self-reported PLS that are worth considering. Specifically, the items *“I show a desire to lead, but allow others to share the leadership role”* and “*I am confident regarding personal ideas and beliefs, but acknowledge that I can learn from others*” both primarily loaded onto the Demanding/Flexible factor, not the Self-centred/Other-centred factor as originally proposed by the PLS (Zhang et al., 2014). Western cultures often emphasise individualism, whereas Eastern cultures emphasise collectivism (Hofstede and Bond, 1988; Nisbett et al., 2001). Therefore, managers in Western cultures may perceive the act of asserting their desire to lead and asserting their personal ideas and beliefs as demanding conformity to their way of doing things, rather than being self-centred. They may also perceive the act of allowing others to contribute and lead as being flexible in their approach, rather than being other-centred. Furthermore, the remaining items on the Self-centred/Other-centred factor concern social and relational systems (e.g., *I like to be the centre of attention, but allow others to share the spotlight as well*), whereas the items that loaded onto the Demanding/Flexible factor are more behavioural facets, such as how managers share decisional control in different circumstances (Franken et al., 2020). Another item which loaded onto the Demanding/Flexible factor was “*I recognize the distinction between supervisors and subordinates, but do not act superior in my leadership role*.” Therefore, the Demanding/Flexible factor includes aspects of both participation/process directiveness (autocratic-democratic) and direction/outcome directiveness (permissive-directive) in people management (Muczyk and Reimann, 1987; Peterson, 1997). Previous studies of the PLS in Western cultures similarly found that the Demanding/Flexible factor was not supported due to a high number of factor cross-loadings and poor content adequacy (Franken et al., 2020; Shi, 2018), indicating that leadership behaviours related to controlling processes and outcomes may be somewhat overlapping and interrelated in Western cultures and more difficult to delineate compared to Eastern cultures. The sports industry is inherently marked by paradox and tension, requiring leaders to navigate competing demands while maintaining strategic coherence. The blurring of process and outcome control in paradoxical leadership behaviours may reflect the unique pressures of this fast-paced and high-stakes environment, where uncertainty and competition necessitate integrated strategies. This challenge is particularly evident in contexts such as managing elite athletes, launching a sports tech product, or broadcasting a sporting event to a global audience—scenarios in which leaders often conflate these dimensions to address volatility effectively. For example, tensions arise between high-performance sport and mass participation (Guevara-Pérez et al., 2022; Spaaij et al., 2019), balancing athlete well-being with elite performance demands (Holden et al., 2025), and maintaining organisational values while adhering to operational realities (Bell-Laroche et al., 2014; Kerwin et al., 2014). Governance complexities further intensify these paradoxes, as sport organisations must reconcile competing stakeholder interests, financial sustainability, and social responsibilities, often leading to role ambiguity and ethical dilemmas (Clune et al., 2019; De Bosscher and Sotiriadou, 2019; English et al., 2021). A strength of the current study was the use of ESEM which allows for cross-loadings, which may better account for the theoretical complexity of paradoxical leadership behaviours, and more accurately model a more diffuse factor structure resultant from examining paradoxical leadership behaviours across diverse contexts and cultures. Nevertheless, future research may consider how modifications can be made to item wording to increase cultural responsiveness towards Western practises and perceptions.

The validation of a self-reported assessment of paradoxical leadership behaviour has practical implications. While ratings from subordinates and supervisors are essential components to feedback, 360-degree feedback dictates that individuals assess themselves (Nowack and Mashihi, 2012). Comparisons between an individual’s self-assessment and the assessment of them by others provides important and rich information in 360-degreee appraisal systems (van der Heijden and Nijhof, 2004). Indeed, simultaneously considering self-ratings and other ratings is important for explaining managerial effectiveness (Atwater et al., 1998). Scholars have argued that self-perception biases, which is the difference between self-perceptions and perceptions of others, is a predictor of organisational and individual performance (Yammarino and Atwater, 1993), and empirical research has demonstrated that high performing managers have substantially less self-perception bias compared to average performing managers (Church, 1997). Leader’s self-perceptions show how they aim to enact leadership (Hartung, 2020) and having a manager self-assess their own leadership behaviours in comparison to others assessment may help them become more self-aware of their behaviours and engage in leadership development programmes to overcome their shortcomings (Dussault et al., 2013). However, the assumption that differences in self-assessment and assessment of others reflects a true difference in perception only holds if the scales used to assess self-perceptions and the perceptions of others are directly comparable. This is particularly salient in VUCA environments, where leaders must dynamically balance competing demands (e.g., flexibility vs. control, empowerment vs. accountability). Paradox theory posits that effective leadership in such contexts requires cognitive and behavioural agility to reconcile tensions (Smith and Lewis, 2011). By providing a tool to measure self-awareness of these behaviours, the scale advances research on how leaders cognitively frame paradoxes and whether alignment between self-and other-ratings predicts resilience in turbulent settings.

The study is also the first to examine paradoxical leadership behaviours in the context of sport industry leadership. As noted previously, research on leadership inside sport has considered many different leadership styles, such as transformational (Peachey et al., 2015) and servant leadership (Hammermeister et al., 2008; Robinson et al., 2018). Yet, in the context of sport, paradoxical or conflicting (contradictory) priorities abound. Most notably, in sport, businesses (e.g., clubs and teams) both collaborate and compete (Chadwick, 2009). As Peachey et al. (2015; p.581) noted, the unique governance and legal structures of sport provide unique leadership challenges to navigate this “cooperation/collaboration dynamic.” As noted previously, this is an exemplar to the fact that paradoxes are not only about contradictions, but they are also interrelated and persistent (Schad et al., 2016). For example, at the grass-roots level, sporting organisations must balance growing the sport and ensuring financial stability (Clune et al., 2019; De Bosscher and Sotiriadou, 2019; English et al., 2021), sports tech organisations must be able to adapt to change to stay competitive while maintaining core values, and sports media organisations must balance editorial integrity with commercial interests (Boyle, 2017). Our results confirm that sport leaders, across a range of different industries and countries, practice variable paradoxical leadership behaviours in a manner consistent with a model theorised from outside sport (Zhang et al., 2014), albeit with minor adjustments. Importantly, a validated self-report scale could be useful in future research, especially in relation to examining the psychological antecedents of paradoxical leadership across different contexts of sport. As noted earlier, there is a dearth of evidence regarding the individual-level factors associated with paradoxical leadership (Batool et al., 2023; Lee et al., 2023). Sport could be an especially fruitful context from which to explore these relationships. Sports organisations are inherently dynamic environments where leadership demands often include balancing competing interests, such as cooperation versus competition and short-term success versus long-term development. The unique pressures and high visibility of sport create a rich environment for studying paradoxical leadership. Moreover, given the inherent governance structure of sport—the cooperation-collaboration paradox—understanding how paradoxical leadership influences organisational outcomes in sport is important area of further inquiry. A self-report may provide useful alongside a subordinate-reported measure to better understand this leadership style, inside this context. At a more practical level, a validated self-report scale for sport leaders could have valuable applications at the individual and organisational levels. Regarding the latter, the scale could help identify individual leaders’ strengths and areas for improvement to provide tailor-made mentorship when an organisation is facing complex challenges. Organisationally, using the scale could help make decisions on the type of upskilling programmes offered to leaders. For instance, if, on average, leaders of an organisation rate themselves low in balancing enforcement vs. flexibility, specific training could be provided to help them develop strategies to recognise when a situation needs one or the other and adapt their behaviours accordingly.

An important finding of this study that adds to previous research is that the factorial structure of the self-reported version of the PLS was invariant across a range of personal and contextual factors including the participant managerial level, years of experience, gender and age. The only caveat to this finding was that some items relating to control and empowerment differed as a function of age. This may reflect the impact of the dynamics of younger-supervisor-older-subordinate relationships, and vice-versa, and stereotypes which may exist in these relationships. For example, young supervisors may believe that that older subordinates may resist their leadership (Smith and Harrington, 1994) or feel reluctant to give orders to subordinates who are much older than them (Collins et al., 2009). Older managers may have the commonly held belief that younger subordinates may hold engage in more innovative-related behaviours (Ng and Feldman, 2013) and therefore lean on this expertise more regularly. The finding that the scale was invariant across a range of individual factors suggests that the scale’s robustness and applicability across diverse managerial and organisational contexts, provides a reliable tool for assessing paradoxical leadership behaviours across entire organisations. A practical outcome of this finding is the ability to compare both the presence and the impact of paradoxical leadership across a different settings and industries, especially in the context of sport. Future research should examine how paradoxical behaviour may be differentially associated with different organisational outcomes (e.g., innovation, employee performance) in different settings and across different leader demographics and characteristics. Of note, the results did support invariance across genders, although these results are based on a highly imbalanced sample, where three-quarters of respondents were men. This reflects broader gender inequities that persist in sporting organisations where men continue to hold the most senior decision-making roles (Burton, 2015; Evans and Pfister, 2020). Additionally, although the results from the study did demonstrate measurement invariance across geographic regions, these results need to be interpreted carefully. Specifically, despite aiming to recruit a diverse international sample, participants from Europe are highly over-represented (58% of respondents were located in Europe), especially in comparison to participants located in South America (2% of respondents were from South America) and Africa (4% of respondents were located in Africa). Therefore, although the scale certainly has utility in diverse settings, whether the paradoxical leadership scale offers a universal metric for researchers and practitioners across the globe aiming to understand and implement paradoxical leadership principles effectively remains somewhat unclear. Further research on the validity of the paradoxical leadership scale in a wide range of geographic locations in imperative to examine how leaders from diverse cultural backgrounds apply paradoxical leadership principles. Comparing how paradoxical leadership influences team cohesion and performance in a professional football club in Europe versus a basketball team in the United States, or how it affects innovation in coaching strategies within elite training centres in Australia compared to grassroots sports development programmes in Africa, offer other potential avenues of inquiry, for example. Finally, it could be used to explore how paradoxical leadership impacts the growth of sport tech start-ups in Silicon Valley or the operational success of sports apparel companies in Asia. Although sporting organisations provide an interesting case study, of course, paradoxical leadership is not exclusive to sports; therefore, future studies should explore how the self-reported scale performs in other industries outside of sports to increase its applicability.

The emphasis on transformational and servant leadership in sports reflects a broader trend within leadership studies, where the vision and motivational prowess of transformational leaders are often heralded as key drivers of team performance and organisational success (Peachey et al., 2015). Similarly, servant leadership, with its focus on the growth and well-being of teams and communities, aligns well with the ethos of sports organisations that value community engagement and team development (Hammermeister et al., 2008). However, this focus may overlook the nuanced complexities and demands of the sports industry, where leaders frequently navigate paradoxical challenges that require balancing competing priorities. For instance, a transformational leadership approach might be most effective in rallying a team during a high-pressure championship run, while servant leadership could excel in youth sports programmes that emphasise character development and community involvement. On the other hand, paradoxical leadership may prove crucial in professional sports environments where balancing short-term performance with long-term sustainability is key. A comparative analysis of different leadership styles (e.g., transformational, servant and paradoxical) in sport could be used to ascertain which leadership styles are the most effective in specific contexts or circumstances. Through such analyses, the interplay between leadership styles and the unique challenges faced in the sports sector, could help identify tailored, context-sensitive approaches to leadership, which could enhance organisational performance.

Finally, confirming that the self-report measure retains the same factor structure as the original establishes its potential utility within sport organisations (and beyond) to support the development and evaluation of leaders in this specific leadership style. For instance, employing the self-report measure to assess leaders’ perceptions of their own behaviours could be particularly valuable for organisations aiming to foster paradoxical leadership among their managers. Our findings suggest that this version is well-suited for practical applications, particularly in leadership development initiatives that rely on self-evaluation.

### Limitations

Like any study, this one has limitations that future research needs to address. The predominance of male participants in upper management positions and residing in Europe limits the findings’ generalizability across genders, hierarchical levels, and geographic regions. Future research should strive for a more balanced representation by employing random sampling techniques to explore potential variations in paradoxical leadership behaviours across different demographics. However, the male over-representation in our sample reflects the persistent structural imbalance in sports, where women remain significantly underrepresented in senior leadership roles within the industry (Burton, 2015). Therefore, random sampling may need to be supplemented with purposive sampling of women in managerial positions to ensure more gender representative samples in future research of paradoxical leadership behaviours. The degree to which cultural nuances affect the interpretation and practice of paradoxical leadership behaviours is an area ripe for deeper exploration, and a cross-cultural validation of the scale is imperative to ensuring the utility of the scale in global contexts. Employing qualitative methodologies to uncover how cultural factors influence these leadership behaviours is crucial for further research. The current study offers a snapshot of paradoxical leadership behaviours within the sports industry. Yet, understanding how these behaviours evolve, especially in response to significant industry shifts or global events, requires longitudinal studies to track changes in paradoxical leadership behaviours over time. Such research could provide insights into the dynamics of paradoxical leadership as it adapts to changing environments and organisational contexts. Another limitation of the study was the reliance on self-reported data from a single informant on a single occasion. While there is value in validating a self-report measure for sports leaders, leaders may have biased views of their actual abilities and behaviours. It is essential to examine the convergent validity of this scale using subordinate ratings of paradoxical leadership behaviours. Therefore, future could provide stronger findings by collecting data from multiple sources (e.g., examining self-other evaluation agreement). Lastly, although MIMC is a valid methodology for assessing the assumption of invariance with several benefits, it cannot confirm these assumptions (Marsh et al., 2014). Future studies should consider employing multiple-group methodologies to verify measurement invariance in the self-reported version of the PLS.

## Conclusion

This study represents an important advancement in the research and practice of paradoxical leadership within the global sports industry. By validating a self-report version of the PLS (Zhang et al., 2014) and confirming its factorial structure across diverse cultural contexts, our findings provide a strong foundation for further exploration of this leadership style. The demonstrated applicability of paradoxical leadership behaviours across geographical and demographic boundaries highlights their relevance to the complex, dynamic environments of sport organisations. Moreover, the scale’s validation extends its usefulness beyond the realm of sport, providing a versatile tool for studying and applying paradoxical leadership in broader leadership contexts. This foundation not only supports its application in sport but also offers a platform for further examination across industries and leadership settings, showcasing its potential to inform and enhance leadership practices globally.

For researchers, these results open pathways for studying how paradoxical leadership operates within the unique structures and roles of the sport industry, including its influence on team dynamics and organisational outcomes. For practitioners, the validated self-report tool offers a practical resource for leadership development, enabling leaders to reflect on their own behaviours and organisations to foster paradoxical leadership as a strategic approach to navigating competing demands. This study not only broadens the understanding of leadership dynamics in sport but also establishes a platform for continued research and practical applications that can help the industry address its evolving challenges.

## Data Availability

The raw data supporting the conclusions of this article will be made available by the authors, without undue reservation.

## Footnotes

^1^ https://www.sportspromedia.com/

## References

[ref1] Aarons G. A. Ehrhart M. G. Farahnak L. R. Sklar M. Horowitz J. (2017). Discrepancies in leader and follower ratings of transformational leadership: relationship with organizational culture in mental health. Adm. Policy Ment. Health Ment. Health Serv. Res. 44, 480–491. doi: 10.1007/s10488-015-0672-7, PMID: 26164567 PMC4775440

[ref2] Alexander T. Thibault L. Frisby W. (2008). Avoiding separation: sport partner perspectives on a long-term inter-organisational relationship. Int. J. Sport Manag. Mark. 3, 263–280. doi: 10.1504/IJSMM.2008.017192

[ref3] Amason A. C. (1996). Distinguishing the effects of functional and dysfunctional conflict on strategic decision making: resolving a paradox for top management teams. Acad. Manag. J. 39, 123–148. doi: 10.2307/256633

[ref4] Andriopoulos C. Lewis M. W. (2009). Exploitation-exploration tensions and organizational ambidexterity: managing paradoxes of innovation. Organ. Sci. 20, 696–717. doi: 10.1287/orsc.1080.0406, PMID: 19642375

[ref5] Aoki K. (2019). The roles of material artifacts in managing the learning–performance paradox: the kaizen case. Acad. Manag. J. 63, 1266–1299. doi: 10.5465/amj.2017.0967

[ref6] Arthur C. A. Bastardoz N. Eklund R. (2017). Transformational leadership in sport: current status and future directions. Curr. Opin. Psychol. 16, 78–83. doi: 10.1016/j.copsyc.2017.04.001, PMID: 28813361

[ref7] Asparouhov T. Muthén B. (2009). Exploratory structural equation modeling. Struct. Equ. Model. Multidiscip. J. 16, 397–438. doi: 10.1080/10705510903008204

[ref8] Atwater L. E. Ostroff C. Yammarino F. J. Fleenor J. W. (1998). Self-other agreemnt: does it really matter? Pers. Psychol. 51, 577–598. doi: 10.1111/j.1744-6570.1998.tb00252.x

[ref9] Babiak K. Thibault L. (2007). Challenges in multiple cross-sector partnerships. Nonprofit Volunt. Sect. Q. 38, 117–143. doi: 10.1177/0899764008316054

[ref10] Batool U. Raziq M. M. Sarwar N. (2023). The paradox of paradoxical leadership: a multi-level conceptualization. Hum. Resour. Manag. Rev. 33:100983. doi: 10.1016/j.hrmr.2023.100983

[ref11] Bell-Laroche D. MacLean J. Thibault L. Wolfe R. (2014). Leader perceptions of management by values within Canadian national sport organizations. J. Sport Manag. 28, 68–80. doi: 10.1123/jsm.2012-0304

[ref12] Bennis W. G. Naus B. (1985). Leaders: The strategies of taking charge. New York: Harper Row.

[ref1002] Boyle R. (2017). Sports journalism: Changing journalism practice and digital media. Taylor & Francis. 5, 493–495.

[ref13] Browne M. W. (2001). An overview of analytic rotation in exploratory factor analysis. Multivar. Behav. Res. 36, 111–150. doi: 10.1207/S15327906MBR3601_05

[ref14] Burton L. J. (2015). Underrepresentation of women in sport leadership: a review of research. Sport Manag. Rev. 18, 155–165. doi: 10.1016/j.smr.2014.02.004

[ref15] Caniëls M. C. (2023). How perceptual differences between leaders and followers affect the resilience-workability relationship. Front. Psychol. 13:1066909. doi: 10.3389/fpsyg.2022.1066909, PMID: 36698602 PMC9869059

[ref16] Chadwick S. (2009). From outside lane to inside track: sport management research in the twenty-first century. Manag. Decis. 47, 191–203. doi: 10.1108/00251740910929786

[ref17] Chen F. F. (2007). Sensitivity of goodness of fit indexes to lack of measurement invariance. Struct. Equ. Model. Multidiscip. J. 14, 464–504. doi: 10.1080/10705510701301834

[ref18] Church A. H. (1997). Managerial self-awareness in high-performing individuals in organizations. J. Appl. Psychol. 82, 281–292. doi: 10.1037/0021-9010.82.2.281, PMID: 9109286

[ref19] Church A. H. Rotolo C. T. (2010). “The role of the individual in self-assessment for leadership development” in Self-management and leadership development. eds. Rothstein M. G. Burke R. J. (Cheltenham, U.K.: Edward ElgarP ublishing Limited).

[ref20] Clune C. Boomsma R. Pucci R. (2019). The disparate roles of accounting in an amateur sports organisation: the case of logic assimilation in the gaelic athletic association. Account. Audit. Account. J. 32, 1926–1955. doi: 10.1108/AAAJ-06-2018-3523

[ref21] Coker I. Cotterill S. T. Griffin J. (2022). Player perceptions of athlete leadership and leadership development in an english premier league football academy. Asian J. Sport and Exercise Psychol. 2, 182–189. doi: 10.1016/j.ajsep.2021.12.001

[ref22] Collins M. H. Hair J. J. F. Rocco T. S. (2009). The older-worker-younger-supervisor dyad: a test of the reverse pygmalion effect. Hum. Resour. Dev. Q. 20, 21–41. doi: 10.1002/hrdq.20006

[ref23] Conway J. M. Huffcutt A. I. (1997). Psychometric properties of multisource performance ratings: a meta-analysis of subordinate, supervisor, peer, and self-ratings. Hum. Perform. 10, 331–360. doi: 10.1207/s15327043hup1004_2

[ref24] De Bosscher V. Sotiriadou P. (2019). “The governance of the board of national governing bodies in high performance sport” in Research handbook on sport governance. eds. Winand M. Anagnostopoulos C. (Cheltenham, U.K.: Edward Elgar Publishing).

[ref25] Dussault M. Frenette É. Fernet C. (2013). Leadership: validation of a self-report scale. Psychol. Rep. 112, 419–436. doi: 10.2466/01.08.PR0.112.2.419-436, PMID: 23833872

[ref26] Ellison L. J. Steelman L. A. Young S. F. Riordan B. G. (2022). Setting the stage: feedback environment improves outcomes for a 360-degree-feedback leader-development program. Consult. Psychol. J. 74, 363–382. doi: 10.1037/cpb0000236, PMID: 39780972

[ref27] English C. Nash C. Martindale R. (2021). Exploring the coach–administrator relationship within the sa cricket development environment. Eur. Sport Manag. Q. 21, 466–483. doi: 10.1080/16184742.2020.1749689

[ref28] Evans A. B. Pfister G. U. (2020). Women in sports leadership: a systematic narrative review. Int. Rev. Sociol. Sport 56, 317–342. doi: 10.1177/1012690220911842, PMID: 39896927

[ref29] Franken E. Plimmer G. Malinen S. (2020). Paradoxical leadership in public sector organisations: its role in fostering employee resilience. Aust. J. Public Adm. 79, 93–110. doi: 10.1111/1467-8500.12396

[ref30] Guevara-Pérez J. C. Rojo-Ramos J. Urdaneta-Camacho R. Vallespín E. M. (2022). Raiders of the olympic values: perception of the development of women’s canoeing in Spain for Tokyo 2021. Int. J. Environ. Res. Public Health 19:6909. doi: 10.3390/ijerph19116909, PMID: 35682492 PMC9180230

[ref31] Hammermeister J. Burton D. Pickering M. Chase M. Westre K. Baldwin N. (2008). Servant-leadership in sport: a concept whose time has arrived. Int. J. Servant-Leadership 4, 185–215. doi: 10.33972/ijsl.243

[ref32] Harris M. M. Schaubroeck J. (1988). A meta-analysis of self-supervisor, self-peer, and peer-supervisor ratings. Pers. Psychol. 41, 43–62. doi: 10.1111/j.1744-6570.1988.tb00631.x

[ref33] Hartung P. (2020). The impact of self-awareness on leadership behavior. J. App. Leadership Manag. 8, 1–21.

[ref34] Hofstede G. Bond M. H. (1988). The confucius connection: from cultural roots to economic growth. Organ. Dyn. 16, 5–21. doi: 10.1016/0090-2616(88)90009-5

[ref35] Holden J. Wagstaff C. R. D. Wadey R. Brown P. (2025). Navigating athlete development in elite sport: understanding the barriers to the provision of performance lifestyle service in England. Psychol. Sport Exerc. 77:102779. doi: 10.1016/j.psychsport.2024.102779, PMID: 39551251

[ref36] Hu L. T. Bentler P. M. (1999). Cutoff criteria for fit indexes in covariance structure analysis: conventional criteria versus new alternatives. Struct. Equ. Model. Multidiscip. J. 6, 1–55. doi: 10.1080/10705519909540118

[ref37] Isard R. F. Melton E. N. Delia E. B. Nite C. (2023). Between profit and purpose: employee responses to financial and social logics in women’s sport. J. Sport Manag. 38, 153–167. doi: 10.1123/jsm.2022-0344, PMID: 36626690

[ref38] Ishaq E. Bashir S. Khan A. K. (2021). Paradoxical leader behaviors: leader personality and follower outcomes. Appl. Psychol. 70, 342–357. doi: 10.1111/apps.12233

[ref39] Keller J. Loewenstein J. Yan J. (2017). Culture, conditions and paradoxical frames. Organ. Stud. 38, 539–560. doi: 10.1177/0170840616685590

[ref40] Kerwin S. MacLean J. Bell-Laroche D. (2014). The mediating influence of management by values in nonprofit sport organizations. J. Sport Manag. 28, 646–656. doi: 10.1123/JSM.2013-0303

[ref41] Kim H.-D. Cruz A. B. (2016). The influence of coaches’ leadership styles on athletes’ satisfaction and team cohesion: a meta-analytic approach. Int. J. Sports Sci. Coach. 11, 900–909. doi: 10.1177/1747954116676117

[ref42] Lee M. Jung M. (2022). The mediating effect of empathy between mindfulness and self-leadership in female university students: a cross-sectional study. Int. J. Environ. Res. Public Health 19:15623. doi: 10.3390/ijerph192315623, PMID: 36497695 PMC9736619

[ref43] Lee A. Lyubovnikova J. Zheng Y. Li Z. F. (2023). Paradoxical leadership: a meta-analytical review. Front. Organizational Psychol. 1:1229543. doi: 10.3389/forgp.2023.1229543

[ref44] Lewis M. W. (2000). Exploring paradox: toward a more comprehensive guide. Acad. Manag. Rev. 25, 760–776. doi: 10.2307/259204

[ref45] Lewis M. W. Andriopoulos C. Smith W. K. (2014). Paradoxical leadership to enable strategic agility. Calif. Manag. Rev. 56, 58–77. doi: 10.1525/cmr.2014.56.3.58

[ref46] Lewis M. W. Smith W. K. (2022). Reflections on the 2021 amr decade award: navigating paradox is paradoxical. Acad. Manag. Rev. 47, 528–548. doi: 10.5465/amr.2022.0251

[ref47] Li P. P. (2016). Global implications of the indigenous epistemological system from the east. Cross Cultural Strat. Manag. 23, 42–77. doi: 10.1108/CCSM-10-2015-0137

[ref48] Marsh H. W. Morin A. J. S. Parker P. D. Kaur G. (2014). Exploratory structural equation modeling: an integration of the best features of exploratory and confirmatory factor analysis. Annu. Rev. Clin. Psychol. 10, 85–110. doi: 10.1146/annurev-clinpsy-032813-153700, PMID: 24313568

[ref49] Marsh H. W. Muthén B. Asparouhov T. Lüdtke O. Robitzsch A. Morin A. J. S.. (2009). Exploratory structural equation modeling, integrating cfa and efa: application to students' evaluations of university teaching. Struct. Equ. Model. Multidiscip. J. 16, 439–476. doi: 10.1080/10705510903008220

[ref50] Miron-Spektor E. Ingram A. Keller J. Smith W. K. Lewis M. W. (2017). Microfoundations of organizational paradox: the problem is how we think about the problem. Acad. Manag. J. 61, 26–45. doi: 10.5465/amj.2016.0594

[ref51] Morin A. J. S. Arens A. K. Marsh H. W. (2016). A bifactor exploratory structural equation modeling framework for the identification of distinct sources of construct-relevant psychometric multidimensionality. Struct. Equ. Model. Multidiscip. J. 23, 116–139. doi: 10.1080/10705511.2014.961800

[ref52] Muczyk J. P. Reimann B. C. (1987). The case for directive leadership. Acad. Manag. Exec. 1, 301–311. doi: 10.5465/ame.1987.4275646, PMID: 39454232

[ref53] Murphy K. R. (2008). Explaining the weak relationship between job performance and ratings of job performance. Ind. Organ. Psychol. 1, 148–160. doi: 10.1111/j.1754-9434.2008.00030.x

[ref54] Ng T. W. H. Feldman D. C. (2013). A meta-analysis of the relationships of age and tenure with innovation-related behaviour. J. Occup. Organ. Psychol. 86, 585–616. doi: 10.1111/joop.12031

[ref55] Nisbett R. E. Peng K. Choi I. Norenzayan A. (2001). Culture and systems of thought: holistic versus analytic cognition. Psychol. Rev. 108, 291–310. doi: 10.1037/0033-295X.108.2.291, PMID: 11381831

[ref56] Nöthel S. Nübold A. Uitdewilligen S. Schepers J. Hülsheger U. (2023). Development and validation of the adaptive leadership behavior scale (albs). Front. Psychol. 14:1149371. doi: 10.3389/fpsyg.2023.1149371, PMID: 37829081 PMC10565815

[ref57] Nowack K. M. Mashihi S. (2012). Evidence-based answers to 15 questions about leveraging 360-degree feedback. Consulting Psychol. J.: Prac. Res. 64, 157–182. doi: 10.1037/a0030011

[ref58] Peachey J. W. Zhou Y. Damon Z. J. Burton L. J. (2015). Forty years of leadership research in sport management: a review, synthesis, and conceptual framework. J. Sport Manag. 29, 570–587. doi: 10.1123/jsm.2014-0126

[ref59] Peterson R. S. (1997). A directive leadership style in group decision making can be both virtue and vice: evidence from elite and experimental groups. J. Pers. Soc. Psychol. 72, 1107–1121. doi: 10.1037/0022-3514.72.5.1107

[ref60] Raw K. Sherry E. Schulenkorf N. (2022). Managing sport for development: an investigation of tensions and paradox. Sport Manag. Rev. 25, 134–161. doi: 10.1016/j.smr.2020.09.002

[ref61] Robinson G. M. Neubert M. J. Miller G. (2018). Servant leadership in sport: a review, synthesis, and applications for sport management classrooms. Sport Manag. Educ. J. 12, 39–56. doi: 10.1123/smej.2016-0023

[ref62] Rowe W. G. (2001). Creating wealth in organizations: the role of strategic leadership. Acad. Manag. Perspect. 15, 81–94. doi: 10.5465/ame.2001.4251395

[ref63] Schad J. Lewis M. W. Raisch S. Smith W. K. (2016). Paradox research in management science: looking back to move forward. Acad. Manag. Ann. 10, 5–64. doi: 10.5465/19416520.2016.1162422

[ref64] Shi S. (2018). *The cross-cultural generalizability of paradoxical leader behavior theory* [Doctoral dissertation, Hong Kong Polytechnic University]. Polyu Electronic Theses. Available at: https://theses.lib.polyu.edu.hk/handle/200/9674

[ref65] Smith W. J. Harrington K. V. (1994). Younger supervisor-older subordinate dyads: a relationship of cooperation or resistance? Psychol. Rep. 74, 803–812. doi: 10.2466/pr0.1994.74.3.803

[ref66] Smith W. K. Lewis M. W. (2011). Toward a theory of paradox: a dynamic equilibrium model of organizing. Acad. Manag. Rev. 36, 381–403. doi: 10.5465/amr.2009.0223

[ref67] Sotiriadou K. (2009). The australian sport system and its stakeholders: development of cooperative relationships. Sport in Society 12, 842–860. doi: 10.1080/17430430903053067

[ref68] Sotiriadou P. Brouwers J. De Bosscher V. Cuskelly G. (2017). The role of interorganizational relationships on elite athlete development processes. J. Sport Manag. 31, 61–79. doi: 10.1123/jsm.2016-0101

[ref69] Soucie D. (1994). Effective managerial leadership in sport organizations. J. Sport Manag. 8, 1–13. doi: 10.1123/jsm.8.1.1

[ref70] Spaaij R. Lusher D. Jeanes R. Farquharson K. Gorman S. Magee J. (2019). Participation-performance tension and gender affect recreational sports clubs’ engagement with children and young people with diverse backgrounds and abilities. PLoS One 14:e0214537. doi: 10.1371/journal.pone.0214537, PMID: 30995256 PMC6469765

[ref71] Stevens M. Rees T. Cruwys T. (2021). Social identity leadership in sport and exercise: current status and future directions. Psychol. Sport Exerc. 55:101931. doi: 10.1016/j.psychsport.2021.101931

[ref72] van der Heijden B. I. J. M. Nijhof A. H. J. (2004). The value of subjectivity: problems and prospects for 360-degree appraisal systems. Int. J. Hum. Resour. Manag. 15, 493–511. doi: 10.1080/0958519042000181223

[ref73] Vishwakarma S. S. Vishwakarma S. K. (2024). “Agile transformation: business strategies and best practices for vuca and bani world” in Digital project management - strategic theory and practice. ed. Adeyinka-Ojo S.. (IntechOpen).

[ref74] Wareham J. Fox P. B. Cano Giner J. L. (2014). Technology ecosystem governance. Organ. Sci. 25, 1195–1215. doi: 10.1287/orsc.2014.0895, PMID: 19642375

[ref75] Yammarino F. J. Atwater L. E. (1993). Understanding self-perception accuracy: implications for human resource management. Hum. Resour. Manag. 32, 231–247. doi: 10.1002/hrm.3930320204

[ref76] Yang Y. Li Z. Liang L. Zhang X. (2021). Why and when paradoxical leader behavior impact employee creativity: thriving at work and psychological safety. Curr. Psychol. 40, 1911–1922. doi: 10.1007/s12144-018-0095-1

[ref77] Zhang Y. Han Y.-L. (2019). Paradoxical leader behavior in long-term corporate development: antecedents and consequences. Organ. Behav. Hum. Decis. Process. 155, 42–54. doi: 10.1016/j.obhdp.2019.03.007

[ref78] Zhang Y. Waldman D. A. Han Y.-L. Li X.-B. (2014). Paradoxical leader behaviors in people management: antecedents and consequences. Acad. Manag. J. 58, 538–566. doi: 10.5465/amj.2012.0995

[ref79] Zhang M. J. Zhang Y. Law K. S. (2022). Paradoxical leadership and innovation in work teams: the multilevel mediating role of ambidexterity and leader vision as a boundary condition. Acad. Manag. J. 65, 1652–1679. doi: 10.5465/amj.2017.1265

[ref80] Zheng J. Lau P. W. C. Chen S. Dickson G. De Bosscher V. Peng Q. (2019). Interorganisational conflict between national and provincial sport organisations within china’s elite sport system: perspectives from national organisations. Sport Manag. Rev. 22, 667–681. doi: 10.1016/j.smr.2018.10.002

